# A hybrid multi-criteria decision-making framework for prioritising clinical determinants of medical futility in the intensive care unit

**DOI:** 10.1186/s40560-026-00928-w

**Published:** 2026-08-24

**Authors:** Selman Karadayi, Efehan Ulas, Onur Kucuk

**Affiliations:** 1 Department of Anesthesiology and Reanimation, Kirklareli University, Kirklareli, Turkey; 2 Department of Biostatistics and Medical Informatics, Kirklareli University, Kirklareli, Turkey; 3 Department of Anesthesiology and Reanimation, Dokuz Eylul University, Izmir, Turkey

**Keywords:** Medical futility, Intensive care units, Decision making, Multi-criteria decision-making, End-of-life care, Potentially inappropriate treatment

## Abstract

Determinations of medical futility in the intensive care unit (ICU) are high-stakes, ethically charged, and clinically variable. Contemporary literature increasingly frames such situations as potentially inappropriate treatment (PIT), particularly when physiologically feasible interventions are judged unlikely to provide meaningful benefit. This study aimed to construct a transparent decision-analytical framework for identifying the clinical factors ICU physicians prioritise when assessing futility and PIT, using a hybrid multi-criteria decision-making (MCDM) approach. A decision-analytical modelling study was conducted in Türkiye with a national expert panel of 20 ICU physicians with 1–18 years of post-speciality experience. A two-round Delphi pre-process with five intensivists, using an a priori 80% consensus threshold, identified 15 clinical factors and 8 evaluation criteria. Criterion weights were derived using Shannon Entropy, and six MCDM techniques were applied in parallel. Bootstrap resampling, ±20% sensitivity analysis, and Spearman rank correlation were used to assess stability and agreement. Terminal malignancy (F4) and irreversible neurological injury (F5) ranked first and second across all six methods (mean ranks 1.000 and 2.000, *σ* = 0.00, 100% bootstrap stability). Inter-method agreement was very strong (Spearman *ρ* = 0.964–1.000, *p* < 0.001), while inter-group agreement across experience strata was moderate to strong (*ρ* = 0.643–0.807, *p* < 0.01). Documented treatment limitation preference (F15) ranked last across every method and experience group. Early-career clinicians ranked clinical frailty (F2) first, whereas advanced clinicians elevated refractory shock (F6) to fourth. Advanced age (F1) showed the widest experience-related variation, ranking 5th, 14th, and 11th across early-career, mid-career, and advanced groups, respectively. The Top-5 factors remained stable under ± 20% weight perturbation. In this expert-panel MCDM framework, ICU physicians appeared to prioritise biological non-recoverability when assessing futility and PIT, while treating treatment limitation preference as a separate ethical domain. The framework captures clinicians’ cognitive prioritisation rather than outcome-validated prognostic accuracy and should be read as a decision-support scaffold, not a bedside prediction tool. Prospective outcome validation and recalibration across different legal and cultural settings are required before clinical implementation.

**Supplementary Information:**

The online version contains supplementary material available at https://doi.org/10.1186/s40560-026-00928-w.

## Introduction

Survival is an inadequate single measure of success in critical care [1]. Intensive care unit (ICU) patients fall into three trajectories: recovery and step-down to ward care; death despite full physiological support; and a third group who remain ICU-dependent on life-sustaining treatment with an uncertain course. The third trajectory is clinically and ethically distinctive: a substantial proportion of ICU deaths now occur after a deliberate decision to withhold or withdraw treatment rather than after maximal escalation [2]. Thus, clinicians are increasingly required not only to ask whether a patient can be kept alive, but whether ongoing treatment remains beneficial.

The construct traditionally used to organise this question is medical futility. In its canonical formulation, futility refers to an intervention that has no reasonable probability of producing its intended physiological effect, or produces an effect without perceivable benefit to the patient [3]. This includes both a quantitative component concerning physiological recovery and a qualitative component concerning the value of the achievable outcome [4]. Because no universally accepted probability threshold defines either component, the construct therefore retains an irreducibly subjective core in routine practice. To accommodate that subjectivity, five major critical care societies adopted the term potentially inappropriate treatment (PIT) for physiologically feasible interventions deemed inappropriate for clinical, ethical, or pragmatic reasons [5, 6]. Empirical studies confirm that perceived inappropriate care is common and varies substantially between clinicians and healthcare systems [7].

Established prognostic instruments, including the Acute Physiology and Chronic Health Evaluation II (APACHE II) and Sequential Organ Failure Assessment (SOFA), help quantify severity at the cohort level but cannot determine non-benefit for an individual patient [8]. In practice, clinicians integrate multiple domains: baseline reserve, acute physiological trajectory, neurological prognosis, achievable functional outcome, and patient or family values. Futility judgement is, in operational terms, a multi-criteria decision problem.

Multi-criteria decision-making (MCDM) methods provide the mathematical apparatus for that class of problem by formalising criteria, quantifying their relative importance, and ranking alternatives [9]. Although MCDM applications in healthcare have increased substantially [10], to our knowledge, MCDM methodology has not been applied to the prioritisation of futility and PIT determinants in the ICU.

This study applies a hybrid MCDM framework, using a national expert panel of ICU physicians in Türkiye, to identify and rank the clinical factors prioritised when assessing futility and PIT.

## Materials and methods

### Study design and ethics

This was a decision-analytical modelling study conducted in Türkiye, based on anonymised expert ratings collected through a structured survey. No patient-level data, identifiable health information, or clinical interventions were involved. The study was therefore exempt from formal institutional ethics committee review under national research governance criteria for non-interventional methodological work using anonymised expert input.

### Expert panel

Twenty ICU physicians actively practising in critical care were enrolled, spanning anaesthesiology and reanimation, internal medicine intensive care, and emergency medicine intensive care backgrounds, with 1–18 years of completed post-speciality practice. Recruitment used random selection from the eligible national ICU physician registry to maximise heterogeneity of clinical training environments. The panel size was consistent with contemporary healthcare MCDM practice, in which expert panels commonly include approximately 10–25 participants when paired with objective weighting and stability assessment [10]. Anonymity of all individual ratings was preserved throughout collection and analysis to reduce social desirability and authority bias.

For experience-stratified analysis, the panel was a priori divided into three groups: Early-Career (1–5 years), Mid-Career (6–10 years), and Advanced (11–18 years).

### Delphi pre-process for criterion and factor selection

Before the main MCDM analysis, a two-round Delphi consensus process with five senior intensivists was used to derive the criterion and factor sets. The process was conducted and reported in line with the Conducting and REporting of DElphi Studies (CREDES) recommendations [11]. In Round 1, panellists generated and rated candidate clinical factors and evaluation criteria relevant to futility and PIT determination in adult ICU practice. Responses were aggregated, anonymised, and returned as a structured summary. In Round 2, panellists reviewed the aggregated results, revised their ratings where appropriate, and reached consensus through iterative discussion and thematic analysis. Consensus was defined a priori as ≥ 80% agreement on inclusion or exclusion. Where candidate factors showed conceptual overlap, particularly among acute-severity markers that frequently co-occur, items were retained separately only when panellists agreed that they represented distinct bedside decision cues. The final set comprised 8 evaluation criteria (C1–C8) and 15 clinical factors (F1–F15).

### Evaluation criteria and clinical factors

The eight evaluation criteria captured the clinical and ethical dimensions of futility assessment: C1 (Current Clinical Severity), C2 (Mortality Prediction), C3 (Treatment Futility), C4 (Post-Survival Morbidity), C5 (Physiological Reserve), C6 (Communication Potential), C7 (Resource Consumption), and C8 (Human Dignity). Operational definitions are provided in Table 1.

**Table 1:** Evaluation criteria used in the models

Criterion	Evaluation criterion	Clinical focus
C1	Current Clinical Severity	Assessing the acute intensity and gravity of the patient's current medical state
C2	Mortality Prediction	Evaluating the statistical probability that the current ICU stay will end in death
C3	Treatment Futility	Determining if advanced organ supports will fail to yield any meaningful clinical benefit
C4	Post-Survival Morbidity	Estimating the risk of discharge with severe physical or cognitive functional dependency
C5	Physiological Reserve	Identifying signs that the patient's internal recovery capacity and reserves are exhausted
C6	Communication Potential	Assessing barriers to the patient achieving a meaningful level of consciousness or interaction
C7	Resource Consumption	Evaluating the potential for extraordinary use of healthcare resources during and after the ICU
C8	Human Dignity	Evaluating if aggressive treatment conflicts with a death process consistent with human dignity

The 15 clinical factors were grouped into four clinically coherent domains. Chronic Reserve and Baseline Status (F1–F3) comprised advanced age (≥ 80 years), severe frailty (Clinical Frailty Scale [CFS] ≥ 5), and high comorbidity burden (Charlson Comorbidity Index [CCI] ≥ 3) [12–16]. Acute Physiological and Prognostic Severity (F6–F12) included refractory shock, multi-organ failure, severe physiological derangement, severe hypoxaemia, severe metabolic acidosis, requirement for extracorporeal organ support, and failure of SOFA score to decrease within the first 48 h [17–26]. Irreversibility and Treatment Refractoriness (F4, F5, F13, F14) included terminal malignancy, proven irreversible neurological injury excluding brain death, prolonged ICU stay, and prolonged mechanical ventilation with repeated weaning failure [27–30]. The Ethical and Autonomy-Based domain comprised documented treatment limitation preference (F15), defined as a prior patient declaration or refusal of aggressive treatment by the legally designated surrogate or family [31]. Operational definitions of all factors are provided in Table 2.

**Table 2:** Definition of the intensive care futility factors

Factor	Domain	Clinical indicator	Detailed definition
F1	Chronic reserve	Advanced age	Patients aged ≥ 80 years
F2	Chronic reserve	Clinical frailty	Severe loss of baseline functional capacity (CFS ≥ 5)
F3	Chronic reserve	Comorbidity load	Advanced comorbidity burden (CCI ≥ 3)
F4	Irreversibility	Terminal malignancy	Presence of terminal stage malignancy
F5	Irreversibility	Neurological injury	Proven irreversible neurological injury (excluding brain death)
F6	Acute severity	Refractory shock	High-dose vasopressor requirement, tissue hypoperfusion signs, and elevated lactate
F7	Acute severity	Multiple organ failure	SOFA score ≥ 10
F8	Acute severity	Severe physiological derangement	APACHE II score ≥ 25 or GCS score ≤ 8
F9	Acute severity	Respiratory failure	PaO2/FIO2 ≤ 100 mmHg
F10	Acute severity	Metabolic acidosis	Severe metabolic acidosis despite treatment (pH < 7.20)
F11	Acute severity	Advanced organ support	Requirement for extracorporeal organ support (RRT, ECMO)
F12	Acute severity	Clinical trend	Failure to decrease SOFA score within the first 48 h of ICU admission
F13	Irreversibility	Prolonged ICU stay	Length of stay > 21 days
F14	Irreversibility	Ventilation issues	Prolonged MV and repeated weaning failures
F15	Ethical/Autonomy	Treatment limitation preference	Patient’s advance directive or family's refusal of aggressive treatment

### MCDM methods and weighting

Criterion weights Wⱼ were derived using Shannon Entropy, which assigns greater weight to criteria with greater between-alternative variability and lower weight to criteria with more uniform rating distributions. This objective weighting approach reduces the analyst-introduced subjectivity and is widely used in MCDM applications where rating variability is the primary information source [10]. The 9-point Likert ratings were treated as interval-scale variables throughout all weighting and ranking computations. Six MCDM ranking methods were applied in parallel: Technique for Order of Preference by Similarity to Ideal Solution (TOPSIS), Additive Ratio Assessment (ARAS), Complex Proportional Assessment (COPRAS), Weighted Aggregated Sum Product Assessment (WASPAS), Simple Additive Weighting (SAW), and Ranking of Alternatives through Functional Mapping of Criterion Sub-Intervals into a Single Interval (RAFSI). These methods differ in their mathematical assumptions, including distance from ideal solutions, additive utility, proportional assessment, weighted sum-product aggregation, and functional mapping. Using them with different mathematical foundations allowed assessment of whether the resulting ranking was method-dependent or method-invariant.

### Stability and sensitivity analyses

Stability of the ranking was assessed by bootstrap resampling. One thousand iterations were performed with replacement from the 20-expert dataset, and the full MCDM pipeline was re-executed at each iteration. Stability rate was defined as the proportion of iterations in which a factor maintained its observed rank position. MSE of the rank across iterations was calculated as a continuous stability index. Sensitivity to weighting assumptions was tested by perturbing each criterion weight by ± 20% and re-running the pipeline. The coefficient of variation (CV) of rank under perturbation was used to identify factors whose positions were sensitive to weighting choice.

### Statistical analysis

Inter-method agreement across the six MCDM methods and inter-group agreement across the three experience strata were assessed using Spearman’s rank correlation coefficients (*ρ*). Statistical significance was set at *p* < 0.05. Analyses were performed in R version 4.3.1 (R Foundation for Statistical Computing, Vienna, Austria).

## Results

### Shannon Entropy weights

**Fig. 1:**
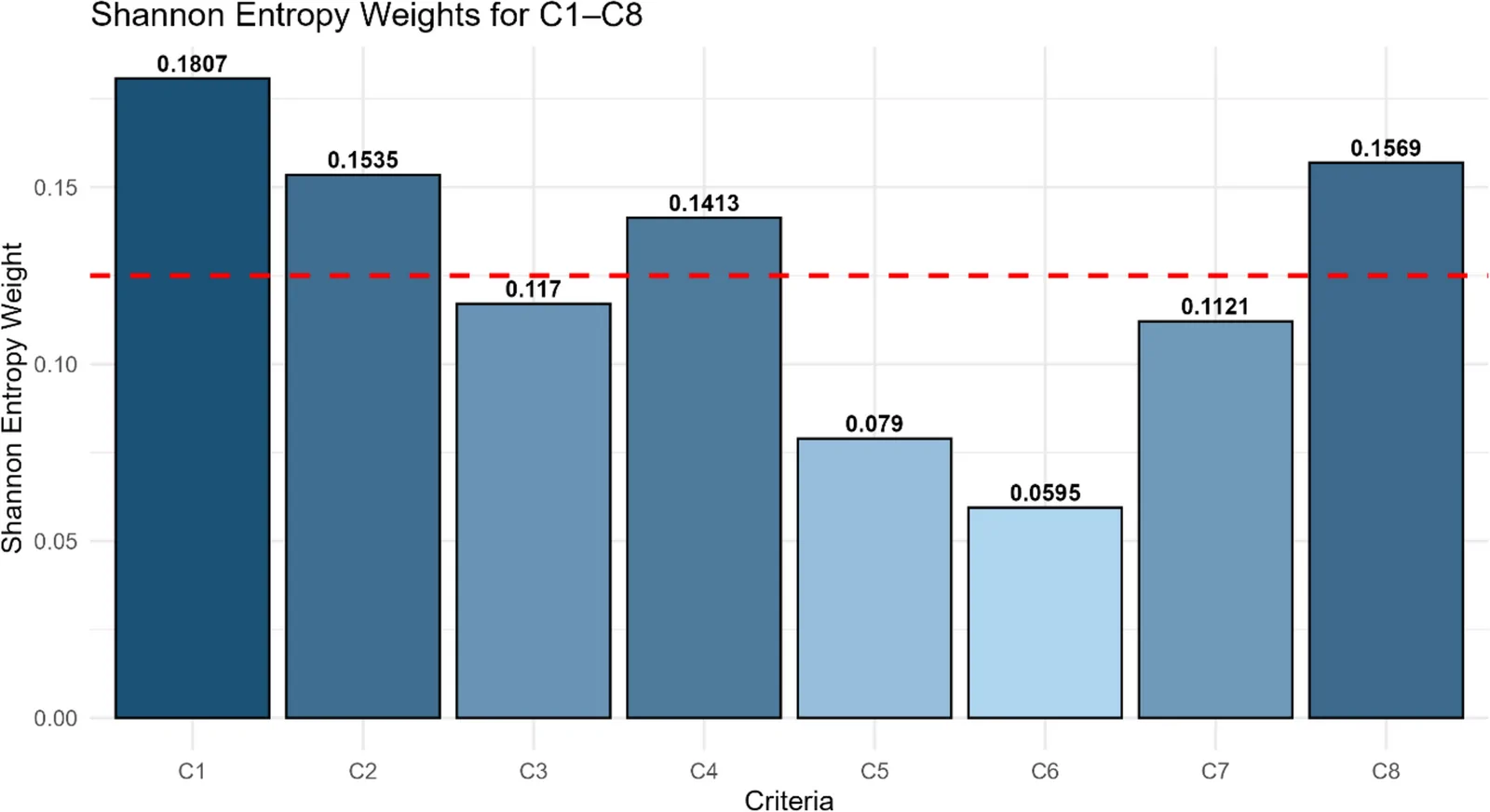
Shannon Entropy weights for C1–C8

Shannon Entropy weights Wⱼ were calculated for the eight evaluation criteria from the variance of expert ratings across the 15 alternatives. Figure 1 shows the Shannon Entropy weights for eight criteria.

### Overall factor ranking

The consolidated rankings across the six MCDM methods are shown in Table 3 and visualised in Figure S1. Terminal malignancy (F4) ranked first across all methods, followed by irreversible neurological injury (F5), with mean ranks of 1.000 and 2.000, respectively, and zero variance. Comorbidity load (F3) ranked third (mean rank 3.000). The mid-tier was occupied by advanced organ support (F11; mean rank 4.167), clinical frailty (F2; mean rank 4.833), multiple organ failure (F7; mean rank 6.000), refractory shock (F6; mean rank 7.333), severe physiological derangement (F8; mean rank 8.333), and severe hypoxemia (F9; mean rank 8.833). Advanced age (F1; mean rank 9.667) and severe metabolic acidosis (F10; mean rank 10.833) occupied the lower-middle range. The bottom of the hierarchy was occupied by prolonged ICU stay (F13; 12.000), prolonged mechanical ventilation (F14; 13.000), failure of SOFA improvement at 48 h (F12; 14.000), and documented treatment limitation preference (F15; 15.000).

**Table 3:**
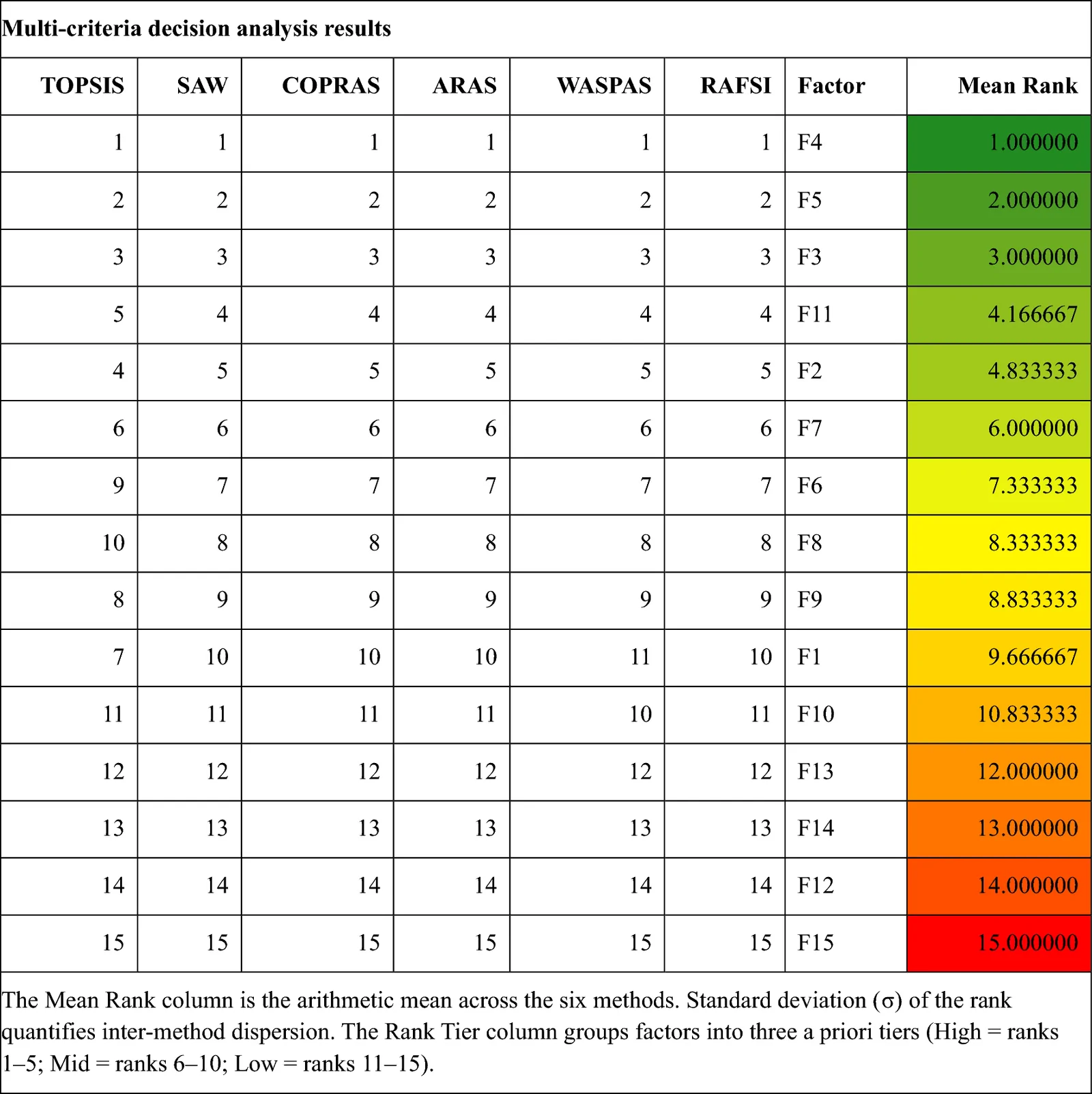
Consolidated clinical factor rankings across MCDM methods

The Mean Rank column is the arithmetic mean across the six methods. Standard deviation (*σ*) of the rank quantifies inter-method dispersion. The Rank Tier column groups factors into three a priori tiers (High = ranks 1–5; Mid = ranks 6–10; Low = ranks 11–15)

### Inter-method agreement

Spearman’s rank correlation coefficients across the six MCDM methods ranged from 0.964 to 1.000 (*p* < 0.001). The TOPSIS-RAFSI pair returned *ρ* = 0.982. The four additive-ratio methods (ARAS, SAW, WASPAS, COPRAS) produced near-identical rank vectors.

### Bootstrap stability

The bootstrap and sensitivity analysis were carried out using RAFSI approach. Across 1,000 bootstrap resampling iterations, F4 and F5 retained their first and second positions in 100% of simulations. Mid-tier factors F7 and F6 maintained rank consistency above 92%; minor rank shifts within this tier were limited to adjacent positions, most frequently observed between multiple organ failure (F7) and clinical frailty (F2) without crossing tier boundaries. The MSE remained below 0.45 for all 15 factors. Bootstrap iteration results are shown in Figure S2.

### Experience-stratified analysis

Experience-stratified rankings are shown in Table 4. Inter-group Spearman correlations ranged from 0.643 to 0.807 (*p* < 0.01). F15 ranked last across all three experience groups. In the Advanced group, F4 and F5 ranked first and second, respectively. In the Mid-Career group, F4 ranked first, whereas F5 ranked third after advanced organ support (F11). In the Early-Career group, clinical frailty (F2) and comorbidity load (F3) ranked first and second, followed by F4 and F5. Three divergences from the consolidated ranking were notable: early-career clinicians ranked frailty (F2) first; advanced clinicians elevated refractory shock (F6) to fourth; and advanced age (F1) showed the widest inter-group spread, ranking fifth, fourteenth, and eleventh in the Early-Career, Mid-Career, and Advanced groups, respectively. The inter-group correlation structure is shown in Figure S3.

**Table 4:** Experience-stratified clinical factor rankings

Factor	Early_Career	Mid_Career	Advanced
F1	5	14	11
F2	1	11	7
F3	2	5	5
F4	3	1	1
F5	4	3	2
F6	12	6	4
F7	7	4	3
F8	9	7	9
F9	8	9	12
F10	10	8	14
F11	6	2	6
F12	14	10	13
F13	11	13	10
F14	13	12	8
F15	15	15	15

Early-Career: 1–5 years; Mid-Career: 6–10 years; Advanced: 11–18 years

### Sensitivity analysis and Pareto decomposition

Under ±20% perturbation of criterion weights, the Top-5 factors (F4, F5, F3, F11, and F2) remained stable, with CV values below 15%. In contrast, the Bottom-5 factors showed greater rank volatility, with larger shifts in response to weighting perturbation. A sensitivity coefficient (Si) was additionally computed for each factor to quantify rank response to unit change in criterion weight; Top-5 Si values fell within the low-response range consistent with the CV finding, whereas Bottom-5 Si values were distributed across the moderate-to-high response range.

**Fig. 2:**
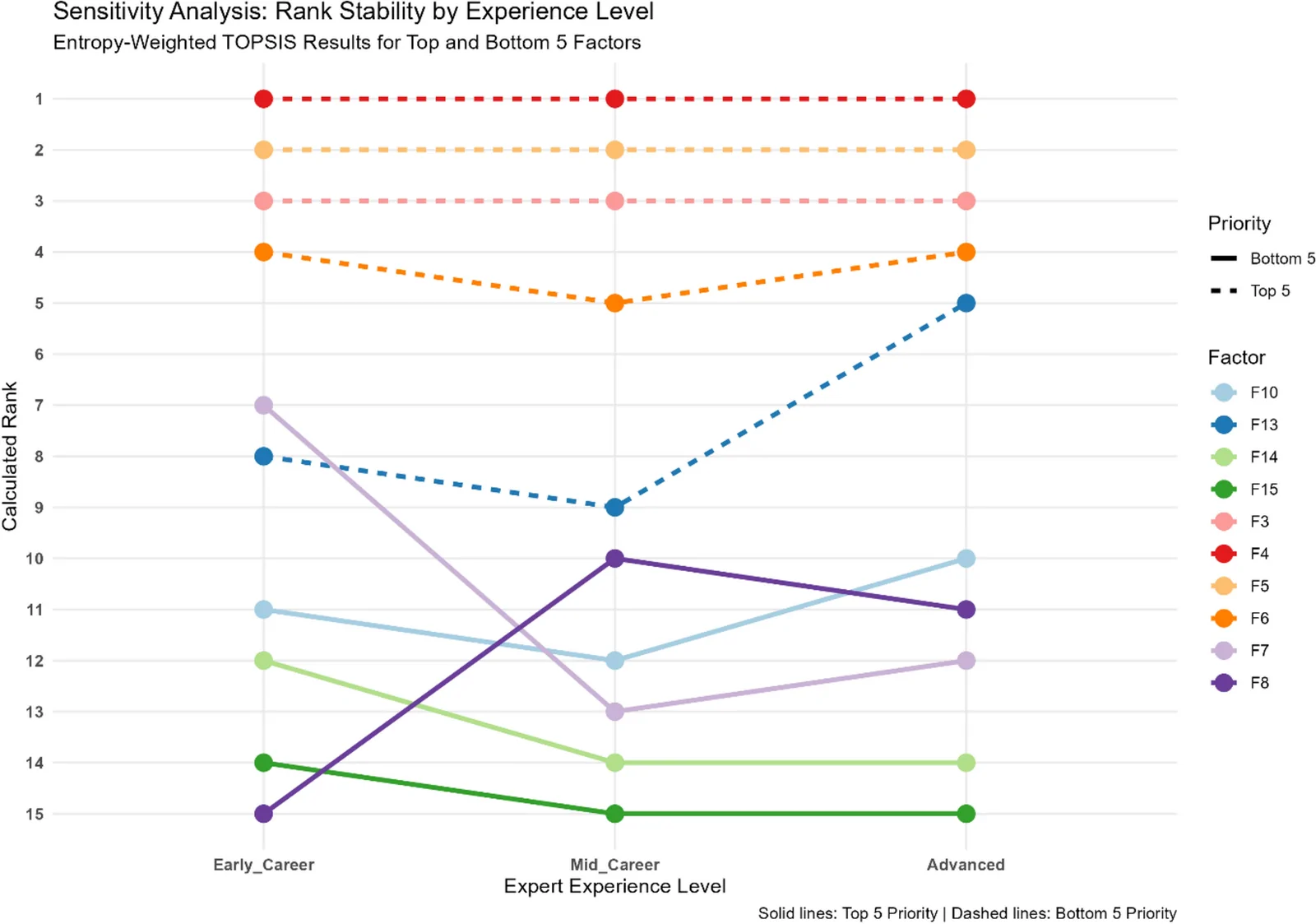
Sensitivity analysis of the top and bottom five factors

Pareto analysis after Z-score normalisation indicated that the Top-5 factors accounted for approximately 80% of the total model contribution. Most Top-5 factors carried Shannon Entropy weights Wⱼ > 0.08, reinforcing their role as the principal informational contributor of the ranking. Sensitivity analysis results are shown in Figs. 2 and S4.

## Discussion

Three principal findings emerge from this analysis. First, terminal malignancy and irreversible neurological injury anchored the consolidated ranking, remaining first and second across all six MCDM methods and 1,000 bootstrap iterations, while remaining within the highest-priority cluster across experience strata. Second, rank position differed between early-career and advanced clinicians in a pattern mapping onto established models of clinical reasoning, most visibly for advanced age and refractory shock. Third, documented treatment limitation preference ranked lowest in every analysis, without exception. Together, these findings indicate that ICU physicians prioritise non-recoverability when judging futility, while treating patient or surrogate preference as a related but separate ethical domain.

The dominant position of terminal malignancy and irreversible neurological injury is consistent with the conceptual foundations of medical futility. Classical definitions distinguish interventions unlikely to achieve their intended physiological objective from those that produce a physiological effect without a meaningful outcome for the patient [3, 4]. Prior qualitative work has similarly identified advanced malignancy, irreversible dependence on life-sustaining treatment, and severe brain injury as prototypical situations in which clinicians invoke futility [32]. Outcome data support this: in a Spanish cohort, patients with solid organ malignancies had 1-year mortality of 75% [27]. Turkish multicentre data point in the same direction: specialist oncology ICUs showed the highest median mortality, and malignancy was the strongest predictor of unit-level death rates [33]. The neurological component is supported by converging evidence: in the Ugandan cohort, GCS ≤ 8 was independently associated with increased mortality [22]. In the present framework, the consistent ranking of F4 and F5 across methods adds a decision-analytical layer to prior qualitative work.

The high ranking of irreversible neurological injury exposes a real conceptual tension worth sitting with. Unlike terminal malignancy, irreversible neurological injury short of brain death does not necessarily preclude continued biological survival. So when clinicians rank it nearly as high, there might be evidence of an unobserved underlying mechanism.

Comorbidity burden and clinical frailty ranked within the upper-middle tier, whereas chronological age alone ranked lower. The CFS has been associated with a per-category mortality hazard of 1.30 (95% CI 1.27–1.33) [14], and ICU studies have supported CFS ≥5 as a clinically meaningful threshold [12]. A Portuguese cohort recently reported in-hospital mortality of 76.3% in the frail subgroup [13]. The empirical position on chronological age has shifted substantially: recent consensus suggests that admission and treatment-intensity decisions should not rest solely on chronological age [34]. In an Australian cohort, algorithm-derived biological age outperformed chronological age in predicting hospital mortality [35]. In the present analysis, age appeared to act as a contextual modifier rather than a primary determinant. The experience-stratified results reinforce this interpretation: advanced age showed the widest inter-group variability, ranking fifth among early-career but much lower among more experienced clinicians. This may suggest that clinical experience shifts attention away from easily measurable demographic markers towards more integrated assessments of biological reserve.

Advanced organ support occupied an upper-tier position, reflecting the ambiguous role of technologies such as extracorporeal membrane oxygenation (ECMO) and renal replacement therapy (RRT) in contemporary critical care. Although these interventions are designed as bridges to recovery, they are often deployed near the limits of physiological reserve and are frequently discussed in the literature on potentially inappropriate treatment [5, 6]. Outcome data substantiate the clinical intuition: in a meta-analysis of 25 studies, including 5,896 ECMO patients, concurrent RRT was associated with a pooled mortality of 63.0% and an 81% relative increase in mortality risk compared with ECMO alone [25]. The present ranking likely reflects this bedside association between extracorporeal escalation and a high-mortality trajectory. However, this result should be interpreted cautiously because F11 grouped ECMO and RRT under a single factor, despite their distinct indications and prognostic implications.

By contrast, acute-severity markers such as refractory shock, multi-organ failure, severe physiological derangement, severe hypoxaemia, and metabolic acidosis occupied the middle tiers. These findings suggest that clinicians distinguish severe physiological derangement from intrinsic non-recoverability. Refractory shock, severe acidosis, or multi-organ failure may indicate high mortality risk, but they can also represent dynamic, treatment-responsive states. In a Finnish nationwide registry, individual SOFA components showed different associations with mortality even within the broader construct of organ failure [19], and in the French cohort, pH correction trajectory rather than admission pH was the independent mortality determinant in severe acidaemia [24]. In sepsis, a patient may simultaneously meet criteria for refractory shock, multi-organ failure, and severe acidosis, yet experienced clinicians recognise this as a treatment target. Recent sepsis guidelines retain aggressive multimodal intervention as a core component of management in severe presentations [36]. Thus, the mid-tier position of F6–F10 should not be interpreted as a low prognostic importance, but as evidence that severity alone is insufficient to define futility.

Chronic critical illness is associated with poor long-term outcomes, including reported 1-year mortality of 48–68% and functional independence in fewer than 12% of survivors [37]. However, no single ICU length-of-stay threshold reliably identifies patients at risk of poor long-term outcome [29], and weaning failure is better understood as a distinct evolving clinical state rather than a simple duration marker [30]. Similarly, although an unchanged or increasing SOFA score during the first 48 h has been associated with mortality of 37–60%, a substantial proportion of patients without early SOFA improvement survive [26]. Thus, time- and trend-based variables may inform prognosis, but they may be insufficient as standalone indicators of futility, particularly within an early 48-h window that may be too short to adjudicate individual reversibility.

The low ranking of documented treatment limitation preference is among the most important findings of the study. F15 ranked last across every method, bootstrap iteration, and experience group. This should not be interpreted as disregard for patient autonomy. Rather, it suggests that clinicians conceptually separate futility from preference-sensitive ethical decision-making: a treatment does not become futile merely because it is declined, nor does it become beneficial merely because it is requested [38]. This distinction is consistent with the rationale behind the term PIT, which was introduced to avoid conflating physiological ineffectiveness with value-laden disagreement [5].

The Turkish legal environment renders this separation unusually visible. Because do-not-resuscitate orders and advance directives carry no binding legal status, patient preferences function more as advisory than decisional instruments [31, 39]. International data also show that end-of-life practices vary substantially across legal and cultural environments, and that national legislation is associated with treatment-limitation practice [2, 40]. The low rank of F15 should therefore be viewed as context-dependent rather than a universal ICU standard; in jurisdictions where advance care planning is legally embedded and routinely practised, preference-based factors might plausibly rank higher. This interpretation is consistent with the 2025 SCCM guideline, which treats communication, symptom management, and institutional PIT policies as domains distinct from a single futility calculation, placing advance directives within a parallel preference-sensitive process [41].

Differences across experience strata illuminate the cognitive mechanisms underlying futility judgement. Early-career clinicians ranked frailty first, whereas advanced clinicians elevated refractory shock. One interpretation is cognitive: less experienced clinicians may rely more heavily on explicit, score-based markers, while experienced clinicians draw increasingly on pattern recognition developed through repeated exposure [42]. Frailty is an accessible and protocol-embedded bedside construct [34], whereas refractory shock may be recognised by advanced clinicians as part of a familiar dying trajectory, particularly when high-dose vasopressor requirements and organ failure coexist [18]. For clinicians whose pattern-recognition repertoire is still developing, this explicit, scored marker may serve as a natural anchor; terminal malignancy and irreversible neurological injury, by contrast, require integration of prognosis, trajectory, and clinical context, features that reward consolidated experience. However, an alternative interpretation should also be considered. The lower priority assigned to frailty by advanced clinicians may reflect not only integrative expertise, but also possible desensitisation within a critical-care culture oriented towards acute physiological rescue. Repeated focus on short-term survival and ICU discharge may reduce sensitivity to the long-term functional decline and poor quality of life that frail patients may experience after critical illness. The shift from “scoring a patient” to “recognising a dying process” may therefore involve both maturation in clinical reasoning and the risk of prognostic blind spots.

Several limitations should be acknowledged. First, the framework captures clinician judgement rather than validated prognostic accuracy; it identifies what clinicians prioritise, not what independently predicts mortality or poor outcome. Second, the panel was single-country and modest in size. Although this is compatible with common healthcare MCDM practice [10], generalizability to other healthcare systems is not established. Third, the criterion and factor sets were generated through a five-intensivist Delphi pre-process and have not been externally validated by a broader international panel. Fourth, the 9-point Likert ratings retain subjective elements, despite the use of Shannon Entropy weighting, six parallel MCDM methods, bootstrap analysis, and sensitivity testing. Fifth, the Turkish legal and cultural context may influence not only F15 but also perceptions of age, frailty, prolonged ICU stay, and treatment limitation. The resulting ranking should therefore be understood as a culturally embedded decision ecology requiring external validation and recalibration before transfer to other healthcare systems.

## Conclusion

This hybrid MCDM framework suggests that ICU physicians primarily prioritize terminal malignancy and irreversible neurological injury when assessing medical futility and potentially inappropriate treatment, with frailty and comorbidity serving as secondary determinants. The low ranking of documented treatment limitation preference suggests that clinicians distinguish physiological non-recoverability from preference-sensitive ethical decision-making. The framework describes a decision-support scaffold, not a validated bedside rule, and requires prospective outcome validation and cross-cultural recalibration before clinical implementation.

## Supplementary Information

Supplementary material 1.

Supplementary material 2.

Supplementary material 3.

Supplementary material 4.

## Data Availability

No datasets were generated or analysed during the current study.
