# Supplementary material for: A hybrid multi-criteria decision-making framework for prioritising clinical determinants of medical futility in the intensive care unit
Source: J Intensive Care. 2026 Aug 24;14:74. doi: 10.1186/s40560-026-00928-w (PMC13501846; doi:10.1186/s40560-026-00928-w)
Supplement: Supplementary file 1 [file 40560_2026_928_MOESM1_ESM.docx]

**Figure S1.** Heatmap of the rankings based on the MCDM approaches. Colour scale runs from rank 1 (highest priority, dark) to rank 15 (lowest priority, light). The vertical alignment of colour across the row identifies factors with identical ranks across methods (visible as horizontal monochrome bands for F4, F5, F3, F12, F13, F14, and F15). Inter-method Spearman rank correlation coefficients ranged from 0.964 to 1.000 (p < 0.001).
