# Supplementary material for: A hybrid multi-criteria decision-making framework for prioritising clinical determinants of medical futility in the intensive care unit
Source: J Intensive Care. 2026 Aug 24;14:74. doi: 10.1186/s40560-026-00928-w (PMC13501846; doi:10.1186/s40560-026-00928-w)
Supplement: Supplementary file 2 [file 40560_2026_928_MOESM2_ESM.docx]

**Figure S2.** Results of the bootstrap iterations. The Mean Standard Error of rank remained below 0.45 for all 15 factors. Stability rate was 100% for F4 and F5, and exceeded 92% for F6 and F7.
