# Supplementary material for: A hybrid multi-criteria decision-making framework for prioritising clinical determinants of medical futility in the intensive care unit
Source: J Intensive Care. 2026 Aug 24;14:74. doi: 10.1186/s40560-026-00928-w (PMC13501846; doi:10.1186/s40560-026-00928-w)
Supplement: Supplementary file 3 [file 40560_2026_928_MOESM3_ESM.docx]

**Figure S3.** Correlation heatmap of the rank positions based on professional experience. Cell values represent the Spearman ρ between each pair of groups; the colour scale runs from low correlation (light) to high correlation (dark). Inter-group correlations spanned 0.643 to 0.807 (p < 0.01), corresponding to moderate to strong agreement.
