# Supplementary material for: A hybrid multi-criteria decision-making framework for prioritising clinical determinants of medical futility in the intensive care unit
Source: J Intensive Care. 2026 Aug 24;14:74. doi: 10.1186/s40560-026-00928-w (PMC13501846; doi:10.1186/s40560-026-00928-w)
Supplement: Supplementary file 4 [file 40560_2026_928_MOESM4_ESM.docx]

**Figure S4.** Mean rank of the bottom and top five factors. Error bars represent the bootstrap 95% CI; "mean rank" denotes the arithmetic mean of the rank position across the bootstrap iterations. The Mean Standard Error of rank remained below 0.45 for all factors, indicating low variability around the central rank estimate.
